# The effect of high compared with low dairy consumption on glucose metabolism, insulin sensitivity, and metabolic flexibility in overweight adults: a randomized crossover trial

**DOI:** 10.1093/ajcn/nqz017

**Published:** 2019-04-17

**Authors:** Coby Eelderink, Susan Rietsema, Iris M Y van Vliet, Larissa C Loef, Theo Boer, Martijn Koehorst, Ilja M Nolte, Ralf Westerhuis, Cécile M Singh-Povel, Jan M W Geurts, Eva Corpeleijn, Stephan J L Bakker

**Affiliations:** 1 Department of Internal Medicinecal; 2 Department of Dietetics; 3 Department of Laboratory Medicine; 4 Department of Epidemiology, University Medical Center Groningen, University of Groningen, Groningen, The Netherlands; 5 Dialysis Center Groningen, Groningen, The Netherlands; 6 Department of Nutritional Sciences, FrieslandCampina, Amersfoort, The Netherlands

**Keywords:** dairy, metabolic flexibility, glucose kinetics, overweight, diabetes

## Abstract

**Background:**

Dairy products contain many nutritious components that may benefit metabolic health. There are indications that glucose metabolism and insulin sensitivity, which are generally disturbed in overweight and obese individuals, may improve by increased dairy intake. This may also affect one's metabolic flexibility.

**Objective:**

The aim of this study was to investigate the effects of high compared with low dairy intake on glucose metabolism, insulin sensitivity, and metabolic flexibility in overweight adults (aged 45–65 y).

**Methods:**

In this randomized intervention study, subjects consumed a high- and a low-dairy diet [HDD (5–6 dairy portions) and LDD (≤1 dairy portion), respectively] for 6 wk in a crossover design, with a washout period of 4 wk. Dairy portions were 200 g semi–skimmed yoghurt, 30 g reduced-fat (30+) cheese, and 250 mL semiskimmed milk and buttermilk. After 6 wk, a 75-g oral-glucose-tolerance test (^13^C-labeled) and a subsequent fasting challenge were performed. Metabolic flexibility was studied by determining the respiratory quotient (RQ) using indirect calorimetry. Fasting and postprandial plasma concentrations of glucose and insulin were analyzed. The dual isotope technique enabled calculation of glucose kinetics.

**Results:**

The study was completed by 45 overweight men and postmenopausal women [age 58.9 ± 4.3 y, BMI 27.9 ± 1.9 kg/m^2^ (mean ± SD)]. Fasting RQ and ΔRQ, reflecting metabolic flexibility, did not differ after both diets. Fasting glucose concentrations were similar, whereas fasting insulin concentrations were lower after the LDD (LDD: 8.1 ± 2.8 mU/L; HDD: 8.9 ± 3.3 mU/L; *P* = 0.024). This resulted in a higher HOMA-IR after the HDD (*P* = 0.027). Postprandial glucose and insulin responses as well as glucose kinetics were similar after both diets.

**Conclusions:**

The amount of dairy intake during a 6-wk period had a neutral effect on metabolic flexibility or postprandial glucose metabolism in middle-aged overweight subjects. More trials are needed to study the effects of specific dairy types and to differentiate between metabolic subgroups. This trial was registered at trialregister.nl as NTR4899.

## Introduction

High dairy intake is associated with improved metabolic health, although depending on the amount and type of dairy products consumed. Several cohort studies have reported that high consumption of dairy products was associated with a lower probability of the metabolic syndrome (1–3). Recent observational studies and meta-analyses have shown that intake of dairy products, such as low-fat dairy, cheese, and yoghurt, may positively affect insulin sensitivity (IS) and reduce the risk of developing type 2 diabetes (T2DM) (4–7). A recent dose-response meta-analysis by Gijsbers et al. (8) suggested mainly yoghurt to be beneficial for reducing T2DM incidence. Positive effects may for instance be due to an increased intake of specific micronutrients (9), amino acids (10), or fatty acids (11). However, confirmation of these suggested beneficial metabolic effects of dairy intake by intervention studies seems challenging. Turner et al. (12) recently reviewed weight-stable intervention studies (*n* = 10) on the effect of dairy intake on IS and reported inconclusive results. Notably, in most dairy intervention studies glucose control was not the primary outcome measure or was limited to basic variables like fasting glucose and insulin (13–15). To observe dietary-induced differences in health outcomes in a relatively healthy population, most fasting measurements are relatively insensitive (16). Hence, it was proposed to measure and define health by looking at an individual's ability to cope with a specific challenge (“resilience”), with restoration of homeostasis as a target resultant of various physiological responses (16–18). Metabolic flexibility, the ability to switch back and forth between the 2 major energy substrates—(glucose and fat)—based on availability and need, forms a hallmark example of such a homeostatic system (19). In general, it is considered healthy (metabolically flexible) if there is an increased reliance on fat oxidation during fasting and during sustained exercise, and an increase in glucose oxidation during insulin-stimulated conditions, such as, after feeding (20). Reduced metabolic flexibility is a common denominator of a cluster of diseases associated with the metabolic syndrome, including cardiovascular diseases and diabetes, and is associated with insulin resistance (21). The direct effect of dairy intake on metabolic flexibility per se has rarely been studied. Dairy is nutrient rich and is an important source of multiple micronutrients (22, 23). At a molecular level, metabolic flexibility relies on the configuration of metabolic pathways, which are regulated by metabolic enzymes and transcription factors, many of which closely interact with micronutrients (24–26). It may therefore be hypothesized that dairy consumption beneficially influences metabolic flexibility. A study by Melanson et al. (27) found that a 1-wk dairy-based high-calcium diet increased 24-h fat oxidation under conditions of acute energy deficit (−600 kcal/d) compared with a low-dairy diet, which may indicate improved metabolic flexibility after a high-dairy intervention.

Here, we assessed the effects of 6 wk high or low dairy intake on metabolic flexibility and glucose metabolism by challenging the body with an oral-glucose-tolerance test (OGTT) and a subsequent fasting challenge (for 8 h after glucose intake). The OGTT was enriched with ^13^C-glucose, and a simultaneous infusion of D-[6,6-^2^H_2_]-glucose (“dual isotope technique”) allowed us to determine glucose kinetics: the rate of appearance of exogenous glucose (RaE), the endogenous glucose production (EGP), and the glucose clearance rate (GCR, rate of glucose uptake in tissues). This provided a more complete view on glucose handling than only measuring total blood glucose as the resultant of these fluxes. Importantly, using GCR together with insulin concentrations, IS could be studied in more detail (28, 29).

## Methods

### Subjects

This randomized crossover intervention study (NTR4899) was performed in middle-aged, overweight individuals [age 45–65 y, BMI (in kg/m^2^) ≥25 to ≤30] because of their increased risk of developing insulin resistance and metabolic inflexibility.

Sample size was calculated with a power calculation on our primary endpoint [metabolic flexibility, Δ respiratory quotient (RQ)], using α = 0.05, β = 0.20, and an expected SD of 0.05, aiming to find a minimal difference of 0.03. In anticipation of some possible dropouts (12.5%), 52 men and postmenopausal women (+1 replacement) were included in the study.

Subjects were mainly recruited via local advertisement (2015–2016). A prescreening questionnaire was used to select possible participants for a screening visit. Eligible subjects were low–medium dairy consumers (∼1–3 servings/d) and were used to consuming 3 main meals/d. They were not involved in intensive sports activities more than twice a week and had a relatively stable weight (self-reported fluctuations in body weight <3 kg in the past 3 mo). Criteria for exclusion, as assessed during screening, were diabetes mellitus [based on criteria of the American Diabetes Association (30): fasting glucose ≥7.0 mmol/L, glycated hemoglobin ≥6.5% (48 mmol/mol)] and clinically relevant abnormalities in blood lipids (total cholesterol >8 mmol/L, triglycerides >6 mmol/L, LDL >5.7 mmol/L), hematology [Hb <8.7 mmol/L (men) or <7.5 mmol/L (women)], or markers for liver damage (alanine aminotransferase and aspartate aminotransferase >45 U/L) or kidney damage (urinary albumin:creatinine ratio >30 mg/mmol). In addition, persons using glucose- or lipid-lowering medication, or other medication influencing glucose metabolism, such as corticosteroids or antipsychotic drugs, were excluded. Stable use of blood pressure–lowering medication was allowed. Additional exclusion criteria were blood donation or use of antibiotics in the past 3 mo, gastrointestinal surgery or dysfunction, or the presence of inflammatory diseases, hepatitis, or HIV infection.

Random assignment was based on minimization (using the software Minim, Stephen Evans, Patrick Royston and Simon Day, UK) (31), to ensure minimal differences in important covariables between groups with a different order of the intervention diet (“High-Low” and “Low-High” groups). Subjects that were found eligible after screening were entered into the randomization program, using information on gender (M/F), age (45–55 y or 55–65 y), and BMI (25–27 or 28–30 kg/m^2^). On the day their first intervention period started, they were informed about the allocated start diet by the researcher.

By design it was not possible to blind neither the participants nor the researcher and dieticians to the diet (high or low dairy). Data processing and analysis of samples were done in a blinded fashion, as e.g., plasma samples and raw data were labeled by period number, not by intervention diet.

The study was conducted according to the principles of the Declaration of Helsinki (last amended by the 64th World Medical Association General Assembly, Fortaleza, Brazil, October, 2013) and in accordance with the Medical Research Involving Human Subjects Act (WMO). Approval was obtained from the Medical Ethics Committee of the University Medical Center Groningen, Groningen, The Netherlands (METc 2014/298). Each subject gave written informed consent for the study. They received compensation (€700 + travel costs) for their hours of participation in the study, based on the Dutch minimum wage.

### Experimental design

**FIGURE 1:**
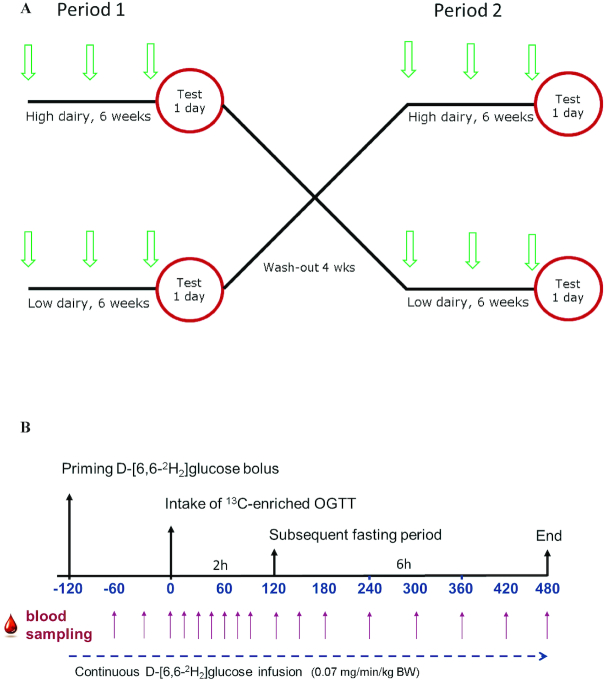
Schematic representation of crossover design (arrows indicate visits) (A) and test day (B). BW, body weight; OGTT, oral-glucose-tolerance test; RQ, respiratory quotient.

Each subject participated in 2 dietary interventions (high and low dairy) for 6 wk in a crossover design, with a washout period in between of 4 wk (**Figure 1**A). After inclusion the subjects came to the hospital on 6 occasions during the 16-wk study period: at the start of each diet (weeks 0 and 10), after 3 wk for a short visit (weeks 3 and 13), and after 6 wk of each intervention for a test day (weeks 6 and 16). During the test day 2 challenge tests were performed to study glucose metabolism and metabolic flexibility: an OGTT as well as a subsequent fasting challenge (Figure 1B). During both tests, respiratory gas exchange was measured allowing the calculation of RQ, as described in detail later. In order to study glucose kinetics and IS, stable isotopes were used; the OGTT was enriched with ^13^C-glucose and subjects received a continuous infusion with a tracer amount of D-[6,6-^2^H_2_]-glucose. To prevent disturbance of the measurements, the subjects were asked to avoid the consumption of ^13^C-enriched foods, like cane sugar, corn products, and pineapple, for 3 d preceding the test day. In addition, subjects had to refrain from alcohol consumption and strenuous exercise for ≥24 h before each study day. To individually standardize food intake each volunteer was asked to prepare and eat the same dinner before both test days. Subjects fasted overnight from 2000 h, but were allowed to drink water. To minimize variation in the 2 fasting RQ measurements performed on the morning of the test day, subjects were instructed to come to the research facility in the most effortless way (e.g., by car instead of bike). After arrival [∼0730, time (*t*) = −150 min], a venous catheter was inserted in each forearm: 1 for blood collection (closed with a 3-way stopcock and flushed with NaCl solution after blood withdrawal) and 1 for infusion of D-[6,6-^2^H_2_]-glucose (98% ^2^H atom% excess) (Isotec). At *t* = −122 min, a total of 26.7 mL D-[6,6-^2^H_2_]-glucose solution (80 × 0.07 mg/kg body weight) was infused within 2 min, and a continuous infusion of 0.07 mg D-[6,6-^2^H_2_]-glucose per kilogram of body weight per minute was started and maintained for 10 h, until *t* = 480 min.

Two hours after the start of the glucose infusion (*t* = 0), the OGTT was started. The OGTT (1.185 atom% ^13^C) consisted of 75 g dextrose (Natufood) dissolved in 300 mL water, with the addition of 0.1% of [U-^13^C]-glucose. To increase palatability by a slight reduction of intense sweetness, 20 drops of lemon juice were added. The glucose drink had to be consumed within 5 min.

During the test day, subjects were on a bed in a semi–upright position and physical activity was limited. Water (150 mL) was provided hourly.

### Dietary intervention

Each subject consumed a high-dairy diet (HDD) and a low-dairy diet (LDD) for 6 wk. At the start of each intervention diet, subjects were instructed by a dietician from the University Medical Center Groningen about the dietary requirements and restrictions. During the HDD, dairy intake consisted of 5 portions for women and 6 portions for men, whereas during the LDD period the subjects consumed ≤1 dairy portion/d. The amount of dairy in both diets was within the range of habitual dairy intake of men or women in the Netherlands [Dutch National Food Consumption Survey 2007–2010 (32)]. The prescribed dairy portions were 200 g semiskimmed yoghurt, 30 g reduced-fat cheese (30+ cheese made from semiskimmed milk containing 30% fat based on dry weight, ∼19 g fat/100 g cheese), and 250 mL semiskimmed milk and/or buttermilk (FrieslandCampina). We chose these basic “medium-fat” dairy products because they are widely used in the Netherlands and represent the average type of dairy used and recommended by our National Guidelines. A standardized cup and bowl were provided. At the start of each intervention period, subjects received a cooled bag of dairy products (with the amount adjusted to the assigned diet). Thereafter, participants bought the prescribed products in their local supermarket.

During the HDD, participants were instructed to consume 1–2 portions of cheese (maturity free of choice) and ≥2 portions of yoghurt each day, whereas the other 1–3 dairy portions could be chosen freely according to preference (mainly milk and/or buttermilk). Except for the prescribed dairy products, all basic and unsweetened, no other dairy products were allowed during the intervention periods, except for the occasional use of small amounts of dairy, e.g., coffee creamer, cooking cream, when used in both intervention periods. During the washout period the volunteers were instructed to return to their habitual diet.

The dairy products were consumed by the volunteers as part of their daily diet. To maintain a stable body weight, caloric intake had to be comparable, so their habitual diet had to be adjusted accordingly, as instructed by the dietician. This was done using a list with common “substitution products” and their approximate caloric value (indicated by “○” representing ∼10 kcal) as guidance. As an example, subjects could see how many slices of bread (plus a certain topping) had a similar amount of calories to a portion of yoghurt (with or without, e.g., cereal or fruit). Compliance to the diet was checked and maintained by regular contact with the dietician and/or researcher (during visits, by phone, and via email) and by dietary assessment (using diaries, as described next). In addition, subjects were asked to hand in the receipts of purchased dairy products to check compliance and for reimbursement of costs.

### Dietary assessment

The subjects registered their intake of dairy portions in a diary on a daily basis. In addition, the subjects were asked to complete a 3-d food diary (for 2 weekdays and 1 weekend day) on 5 occasions: before the start of the study to estimate their habitual diet, and in the third and sixth weeks of both diets. Food diaries were analyzed by a dietitian with the software program EvryDietist, version 6.4.2.1 (Evry BV), which uses the Dutch Food Composition Database 2016 to calculate energy and nutrient intake. The information from week 3 was used to monitor and (if necessary) adjust the subjects’ intake based on their assigned diet and their weight during the visit at week 3, as we aimed for a weight-stable study period.

### Anthropometric measurements

Body weight was measured at each visit with the same calibrated digital measuring scale (Seca 877, Seca GMBH), to monitor whether a stable weight was maintained. Height was measured at week 0 and week 10 (at the start of each diet) using a wall-mounted stadiometer (Seca 222, Seca GMBH). Waist and hip circumference were measured at the start and end of each intervention period, with a measuring tape with a standardized retraction mechanism (Seca 201, Seca GMBH). Measurements were performed in duplicate, without shoes and heavy clothing.

### Body composition

Body composition was estimated using a multifrequency bioelectrical impedance analyzer (QuadScan 4000, BodyStat) on the morning of each test day (weeks 6 and 16). Before the measurement, the subject rested for ≥10 min on the bed in a flat position. Following the manufacturer's instructions, 2 electrodes were placed on the right hand and 2 on the right foot. Although the device records impedance (Z) at 4 frequencies (5 kHz, 50 kHz, 100 kHz, 200 kHz), the 50-kHz impedance was used for calculations using proprietary equations. The bioelectrical impedance analysis was used to estimate body fat percentage and mass (in kilograms), as well as fat-free/lean mass (in kilograms).

### Physical activity

To assess the subject's physical activity during the study period a Baecke questionnaire (33) was completed in the sixth week of each dietary intervention. In addition, the subjects used a pedometer (Digi-walker SW-200, Yamax) during the 3 d preceding the test days, to monitor physical activity. Hereby we could check whether activity was limited and comparable before each test day, and increase subjects’ compliance, because this may influence the RQ results.

### Metabolic flexibility

Metabolic flexibility was assessed by measuring the RQ, as an indicator of substrate oxidation. The RQ is calculated by dividing carbon dioxide production by oxygen consumption. In theory, RQ can have a value between 0.7 (reflecting 100% fat oxidation) and 1.0 (reflecting 100% carbohydrate oxidation). The RQ was determined using the indirect calorimetry system Cosmed Quark RMR (COSMED). The ventilated hood, with a see-through round cap around the head and face, covered subjects’ upper body. They had to lie comfortably and relaxed, and breathe easily in and out. To prevent the volunteers from falling asleep, they were allowed to watch a documentary or movie. During the test day, carbon dioxide concentration in the room was monitored (ATV-Climatrend, ATAL), because increased or fluctuating concentrations could influence the results. To limit variation, all RQ measurements were performed by the same researcher.

The RQ was determined at several time points during the day and each measurement took 30 or 40 min, depending on the time point. Two fasting RQ measurements were performed, from *t* = −90 until *t* = −60 min and from *t* = −45 until *t* = −15 min. The RQ was measured during and after the OGTT at *t* = 5–45, *t* = 65–105, and *t* = 125–165 min, and at *t* = 210–240, *t* = 330–360, and *t* = 435–465 min.

Data from the first and last 5 min of each test were ignored, and the remaining 20 or 30 min were divided into 10-min periods of which a mean RQ value was calculated. This allowed calculation of ΔRQ, which is an indicator of metabolic flexibility, by subtracting the fasting RQ value (RQ before the OGTT—the average of 2 measurements) from the insulin-stimulated RQ value (highest value during/after the OGTT).

### Sample collection

#### Twenty-four-hour urine

Twenty-four-hour urine was collected by the subjects at home the day before the test day, and kept refrigerated. The subjects handed in the containers on the morning of the test day after collecting the last sample, depending on the start time the day before. The volume was calculated by dividing the weight of the urine by 1.015. After centrifugation (2000 × *g* for 10 min at room temperature), aliquots were frozen in 2-mL tubes at −80°C.

#### Blood

In the morning (*t* = −135), fasting blood samples were taken for direct measurements (4.5-mL Lithium-Heparin PST II tube, BD Diagnostics) and for separation of plasma (4-mL EDTA tube and 4-mL Lithium-Heparin tube, BD Diagnostics). During the test days, blood was collected into 2-mL BD Vacutainer Fluoride tubes and 4-mL EDTA tubes (BD Diagnostics). Three basal blood samples were collected (*t* = −60, *t* = −30, *t* = −15 min), then after the OGTT, samples were taken every 15 min for 1.5 h, every 30 min for an additional 1.5 h, and then hourly until *t* = 480. After centrifugation (1300 × *g* for 10 min at room temperature) within 30 min, plasma aliquots were stored at −80°C until analysis.

#### Breath

Breath samples were collected by breathing through a straw into 10-mL Exetainer vials (Labco Limited). Two basal breath samples were collected (*t* = −60, *t* = −5) and after the OGTT a sample was taken every 60 min until *t* = 480.

### Measurements in collected samples

#### Twenty-four-hour urine

Twenty-four-hour urinary excretion of urea and calcium were used as a marker for dietary intake, whereas 24-h urinary creatinine excretion can be used to estimate muscle mass (34) and 24-h urinary albumin excretion can give information about kidney function. Measurements were performed on a Roche/Hitachi Modular automatic analyzer (Roche Diagnostics, Hitachi). In addition, we measured the urinary oxidative stress marker 8-OHdG with an ELISA (Abcam), and malondialdehyde (MDA) using a gas chromatography–mass spectrometry (GC-MS) method as previously described (35).

#### Plasma

Insulin concentrations were measured in plasma (EDTA) on a Roche/Hitachi Modular automatic analyzer (Roche Diagnostics, Hitachi). Plasma glucose concentrations were measured in plasma (NaF) on the same analyzer using a glucose hexokinase method.

In another aliquot (40 µL NaF plasma), derivatization of plasma glucose to glucose penta-acetate was done to enable analysis of isotopic enrichment of orally and intravenously administered D-[U-^13^C]-glucose and D-[6,6-^2^H_2_]-glucose tracers by GC-MS. The sample preparation and measurement were recently described in detail elsewhere (36). GC-MS analyses were performed using positive chemical ionization with ammonia, with ions monitored for *m/z* ranging from *m/z* 408 to 414 (M0–M6). The molar percentage enrichment of [6,6-^2^H_2_]-glucose was calculated using M0 and M2 values, whereas M0 and M6 were used to calculate ^13^C atom%. The obtained values were used for calculation of glucose kinetics.

Blood lipids (total cholesterol, LDL and HDL cholesterol, and triglycerides) and the inflammation marker high-sensitivity C-reactive protein (limit of quantification 0.3 mg/L) were measured on the Roche Modular analyzer directly after blood collection (Li-Hep PST II tube, BD). MDA, as an oxidative stress marker, was analyzed by GC-MS in heparinized plasma.

#### Breath

^13^CO_2_ excretion in breath samples was measured to calculate the oxidation rate of glucose from the OGTT. Analysis of ^13^C abundance in breath carbon dioxide was performed using GC-isotope ratio MS (Delta Plus XL; Thermo Fisher Scientific), measuring the ^13^C/^12^C ratio against the international standard Pee Dee Belemnite (δ^13^CPDB, in ‰), as described previously (37).

### Calculation of glucose kinetics

The rate of appearance of total glucose [RaT, glucose from exogenous (OGTT) and endogenous (hepatic) sources] was calculated from total plasma glucose concentrations and ^2^H-enrichment data by using the non-steady-state equation of Steele et al. (38) as modified by De Bodo et al. (39). It was assumed that labeled and unlabeled glucose molecules showed identical behavior. The effective volume of distribution was assumed to be 200 mL/kg and the pool fraction to be 0.75 (40). The systemic RaE was calculated from the RaT and ^13^C-enrichment data, as described by Tissot et al. (40). The EGP was calculated by subtracting RaE from RaT (40). The GCR, which reflects the tissue glucose uptake, was calculated as described by Schenk et al. (41).

### Incremental AUCs, fasting value, peak value, and time to peak

To determine differences in postprandial plasma glucose, insulin, glucose kinetics, and RQ, the 0–2 h, 0–4 h, and 0–8 h incremental AUCs (iAUCs) were calculated using the trapezoidal rule. The means of fasting measurements (e.g., *t* = −60, −30, −15 for plasma measurements) were used as baseline values, and areas below baseline were not included. Because EGP is suppressed after the OGTT, the area beneath baseline (decremental AUC) was calculated, using the mirrored graphs of this variable. In addition, peak values and time to peak were determined for each variable. For EGP the nadir value and time to nadir are presented. For ^13^CO_2_ the cumulative percentage of the dose was calculated.

### Insulin sensitivity

Basal insulin resistance was estimated by the HOMA-IR, and HOMA-β was used as an indication of β-cell function, both calculated using fasting glucose and insulin concentrations (42).

Whole-body IS was calculated with the Matsuda index using the mean value of glucose and insulin from *t* = 0 to *t* = 120 during the OGTT (43) and mainly peripheral IS by using GCR (iAUC 0–2, 0–4, and 0–8 h) and insulin data [mean postprandial values (without *t* = 0) over the same time period], as previously described (28, 29).

### Well-being and appetite during the test day

Subjective sensation of appetite was assessed ∼15 min before and hourly after the OGTT with a 100-mm visual analogue scale (0 mm stands for no appetite, 100 mm stands for big appetite) until *t* = 480. At the same time points, feeling and extent of discomfort and physical complaints such as abdominal pain, nausea, and headache were scored hourly using a rating system (from 0 = no complaints, to 4 = severe complaints).

### Statistical analyses

Normally distributed variables are reported as mean ± SD and nonnormally distributed variables as median [IQR]. Postprandial data (graphs) are presented as mean ± SEM.

To determine the effect of diet, we used linear mixed-effect models with a random intercept for subject, and with period and diet as fixed factors. Although a washout period of 4 wk, set based on previous reports and practical considerations, was expected to be sufficient, an interaction term period × diet was added to assess a possible carryover effect. A nonsignificant interaction effect was interpreted as the absence of a carryover effect, upon which the term was removed from the model. Log-transformation was applied to normalize skewed data (inflammation and oxidative stress markers; 24-h urinary excretion of albumin, urea, and creatinine; step counter and beverage intake data; and triglycerides) before their use in statistical analyses.

In secondary analyses of our primary endpoint (ΔRQ), relevant covariables (e.g., body weight, caloric intake, macronutrient intake, fiber intake, fasting and peak insulin concentration, or physical activity) were added to the model to adjust for possible confounding. For a selection of outcomes (14 outcomes: fasting RQ; ΔRQ; fasting glucose; fasting insulin; HOMA-IR; HOMA-β; Matsuda index; iAUC 0–4 h of glucose, insulin, RaE, EGP, GCR and RQ; and BMI), secondary analyses were performed adjusting for body weight. In addition, the effect of dietary fiber and beverage intake (coffee, tea, fruit juice, and alcohol) was taken into account in secondary analyses of fasting insulin concentrations, HOMA-IR, and Matsuda index.

For the aforementioned selection of outcomes, we assessed whether the effect of diet was different between metabolically different subgroups. Therefore, additional analyses were performed by adding an interaction term to the model [subgroup (0/1) × diet (0/1)]. We made subgroups based on baseline values of HOMA-IR (above or below 2.56), BMI (above or below 28.2), and fasting glucose [normal fasting glucose (NFG) or impaired fasting glucose (IFG)].

Differences in dietary intake (product groups, **Supplemental Table 1**) were assessed using Wilcoxon's Signed Rank test, because data were nonnormally distributed. All analyses were performed using SPSS 23.0 for Windows (SPSS Inc.). A 2-sided *P* value < 0.05 was considered statistically significant.

## Results

### Baseline characteristics

In total 7 participants dropped out of the study (owing to problems with continuous blood collection). Because the reasons for dropout were unrelated to the diet and there were no data available from the test days, these subjects were excluded from the data analysis. One of the dropouts was replaced, resulting in 46 subjects who completed this study. Data from 1 other participant were excluded, because of illness during the days before the final test day. Exclusion of these dropouts did not affect the results. A flowchart is shown in **Supplemental Figure 1**.

Baseline characteristics (at the start of the first dietary intervention period) of the 45 participants with valid data are shown in **Table 1**.

**TABLE 1:** Baseline characteristics of study participants^1^

Characteristics	
Age, y	58.9 ± 4.3
Gender	
Men, *n* (%)	20 (44.4)
Women, *n* (%)	25 (55.6)
Height, cm	173.7 ± 9.4
Weight, kg	84.3 ± 9.8
BMI, kg/m^2^	27.9 ± 1.9
Waist circumference, cm	95.3 ± 8.7
Hip circumference, cm	107.0 ± 4.5
WHR, cm:cm	0.89 ± 0.08
Glucose, mmol/L	5.6 ± 0.5
Insulin, mU/L	11.1 ± 4.2
HbA1c, %	5.5 ± 0.3
HbA1c, mmol/mol	37.0 ± 3.4
HOMA-IR	2.8 ± 1.2
HOMA-β, %	107.1 ± 41.6
IFG (≥5.6 mmol/L), *n* (%)	22 (48.9)
Cholesterol, mmol/L	5.75 ± 0.96
LDL cholesterol, mmol/L	3.94 ± 0.91
HDL cholesterol, mmol/L	1.55 ± 0.37
Triglycerides, mmol/L	1.11 [0.93–1.81]
Energy intake, kcal/d	2162.7 ± 496.4
Carbohydrate intake, g/d	221.1 ± 65.8
Protein intake, g/d	81.7 ± 18.6
Fat intake, g/d	87.0 ± 24.4
Fiber intake, g/d	21.3 ± 5.1

^1^Values are mean ± SD, median [IQR], or *n* (%), *n* = 45. HbA1c, glycated hemoglobin; HOMA-β, homeostasis model assessment of β cell function; IFG, impaired fasting glucose; WHR, waist-to-hip ratio.

### Dietary intake

Data from the 3-d food diaries completed in weeks 3 and 6 were averaged to get an indication of dietary intake over the 6 wk. When looking at shifts in consumption of the different product groups during both diets (Supplemental Table 1), it was observed that during the LDD there was an increased intake of e.g., beverages such as coffee and tea, pastry, bread, meat products (bread topping), and sweet bread toppings. During the HDD, next to the prescribed dairy products, the use of breakfast cereal was higher.

**Table 2** contains an overview of caloric intake and nutrient intake (per day) during the LDD and HDD. Caloric intake was slightly higher during the HDD. The HDD resulted in a higher intake of mono- and disaccharides (lactose), whereas during the LDD there was a higher intake of polysaccharides and dietary fiber (increased bread intake). During the HDD total protein intake was increased, due to an increased amount of animal protein, whereas plant protein intake was somewhat decreased during the HDD compared with the LDD. Total fat intake was similar between both diets, but during the HDD more saturated fat was consumed. The total amount of *trans* fatty acids (TFAs) in the diets was similar during both diets.

**TABLE 2:** Nutrient intake of 45 overweight men and women during a 6-wk high- or low-dairy diet in a crossover design^1^

	Low-dairy diet	High-dairy diet	*P* value
Energy, kcal/d	2151.7 ± 450.7	2264.2 ± 470.9	0.015
Carbohydrates total, g/d	226.3 ± 55.3	232.0 ± 57.2	0.325
Mono- and disaccharides, g/d	93.6 ± 29.0	113.7 ± 28.4	<0.001
Polysaccharides, g/d	131.9 ± 35.9	118.1 ± 36.2	<0.001
Protein total, g/d	78.8 ± 16.7	105.9 ± 19.2	<0.001
Animal protein, g/d	43.2 ± 14.9	75.5 ± 16.0	<0.001
Plant protein, g/d	35.3 ± 9.3	30.2 ± 7.8	<0.001
Fat total, g/d	82.2 ± 20.2	84.0 ± 19.8	0.513
SFAs, g/d	25.3 ± 7.0	32.3 ± 6.8	<0.001
*Trans* fatty acids (TFAs), g/d	0.71 ± 0.37	0.69 ± 0.26	0.725
MUFAs, g/d	31.7 ± 8.2	28.9 ± 8.1	0.015
PUFAs, g/d	17.9 ± 6.0	14.9 ± 5.3	0.002
Dietary fiber, g/d	22.0 ± 5.4	19.6 ± 5.4	0.001
Calcium, mg/d	706.7 ± 125.2	1955.9 ± 240.9	<0.001
Magnesium, mg/d	356.9 ± 83.3	404.4 ± 73.3	<0.001
Sodium, mg/d	2602.1 ± 654.4	2636.7 ± 609.7	0.717
Potassium, mg/d	3393.9 ± 592.3	4078.9 ± 699.4	<0.001
Zinc, mg/d	10.2 ± 2.2	13.7 ± 2.5	<0.001
Riboflavin, μg/d	1.39 ± 0.48	2.58 ± 0.50	<0.001
Vitamin B-12, μg/d	4.48 ± 2.18	6.96 ± 2.24	<0.001
Vitamin D, μg/d	3.63 ± 1.39	3.05 ± 1.06	0.004

^1^Values are mean ± SD, *n* = 45. Three-day nutritional data of weeks 3 and 6 were averaged. Differences were assessed using linear mixed models.

As a consequence of high dairy consumption, calcium intake was much higher during the HDD. Furthermore, overall micronutrient intake (except vitamin D) increased during the HDD, including magnesium, potassium, zinc, riboflavin, and vitamin B-12 intake (Table 2).

### Physical activity

There was no difference in physical activity during the intervention periods based on the Baecke questionnaire (**Table 3**). The step counter indicated a small difference in activity (∼400 steps) on the day before the test (“day 3”) between the LDD and HDD (*P* = 0.028), but no difference in the average step count during the 3 d preceding both test days.

**TABLE 3:** Physical activity of 45 overweight men and women during a 6-wk high- or low-dairy diet in a crossover design^1^

	Low-dairy diet	High-dairy diet	*P* value
Baecke questionnaire			
Work index	2.49 ± 0.63	2.47 ± 0.65	0.667
Sport index	2.74 ± 0.54	2.75 ± 0.51	0.889
Leisure index	3.17 ± 0.51	3.27 ± 0.57	0.242
Step counter			
Day 1	7624 [5075–9636]	6943 [4152–9822]	0.517
Day 2	5423 [3742–9814]	5581 [4024–8739]	0.976
Day 3	5443 [3213–8821]	5040 [3110–6357]	0.028
Mean steps/day	6621 [4312–8919]	6092 [4488–7787]	0.321

^1^Values are mean ± SD or median [IQR], *n* = 45. The step counter was used during the 3 d preceding the test day (end week 6). Differences were assessed using linear mixed models, with a *P* value < 0.05 considered as statistically significant.

### Body composition and anthropometric measurements

Body weight was somewhat lower after 6 wk of LDD (LDD: 83.7 ± 9.9 compared with HDD: 84.1 ± 9.9 kg; *P* = 0.012). Waist and hip circumference were not different after 6 wk of HDD and LDD (**Table 4**). In addition, bioelectrical impedance analysis measurements of fat percentage and lean mass were not significantly different between the dietary interventions, whereas a trend towards higher fat mass after the HDD was observed (*P* = 0.062).

**TABLE 4:** Subject characteristics and 24-h urinary excretions at the end of 6 wk of low- and high-dairy diet^1^

	Low-dairy diet	High-dairy diet	*P* value
Body composition			
Height, cm	173.7 ± 9.4	173.7 ± 9.4	0.961
Weight, kg	83.7 ± 9.9	84.1 ± 9.9	0.012
BMI, kg/m^2^	27.7 ± 1.9	27.8 ± 1.9	0.009
Waist circumference, cm	94.9 ± 8.7	95.3 ± 8.8	0.218
Hip circumference, cm	106.1 ± 5.0	106.2 ± 5.3	0.787
WHR, cm:cm	0.90 ± 0.08	0.90 ± 0.09	0.304
Fat percentage, %	34.4 ± 8.1	34.6 ± 8.2	0.221
Fat mass, kg	28.4 ± 5.7	28.9 ± 6.3	0.062
Lean mass, kg	55.3 ± 11.7	55.6 ± 11.4	0.622
Blood lipids			
Cholesterol, mmol/L	5.29 ± 0.93	5.26 ± 0.77	0.681
LDL cholesterol, mmol/L	3.55 ± 0.81	3.54 ± 0.34	0.797
HDL cholesterol, mmol/L	1.46 ± 0.38	1.40 ± 0.34	0.002
Triglycerides, mmol/L	1.02 [0.86–1.35]	1.10 [0.94–1.39]	0.066
24-h urine excretion			
Albumin, mg/24 h	4.0 [2.4–6.8]	4.0 [2.3–7.0]	0.657
Urea, mmol/24 h	358.5 [279.1–437.6]	461.7 [392.9–551.3]	<0.001
Creatinine, mmol/24 h	11.6 [9.9–14.8]	11.3 [9.5–14.5]	0.239
Calcium, mmol/24 h	4.1 ± 1.7	4.9 ± 2.4	0.001
Malondialdehyde, µmol/24 h	3.04 [2.55–4.12]	4.38 [3.18–6.56]	0.001
8-OHdG, µg/24 h	408.9 [353.1–478.7]	400.0 [372.3–478.5]	0.745

^1^Values are means ± SDs or median [IQR], *n* = 45. Differences were assessed using linear mixed models, a *P* value < 0.05 was considered statistically significant. WHR, waist-to-hip ratio; 8-OHdG, 8-hydroxy-2-deoxyguanosine.

### Blood lipids

Fasting triglyceride, total cholesterol, and LDL-cholesterol concentrations did not differ between the HDD and LDD (Table 4). HDL-cholesterol concentration was higher after the LDD (*P* = 0.002). Consequently, the cholesterol:HDL ratio was lower after the LDD (LDD: 3.84 ± 1.10; HDD: 3.96 ± 1.07; *P* = 0.015).

### Twenty-four-hour urine excretions of albumin, creatinine, urea, and calcium

Albumin excretion in 24-h urine was the same after both dietary interventions. Twenty-four-hour urinary excretion of creatinine, as a measure of muscle mass, did not differ between the diets. Twenty-four-hour urinary excretions of urea and calcium were higher after the HDD (Table 4), as expected owing to increased intake of protein and calcium from the diet.

### Metabolic flexibility

**FIGURE 2:**
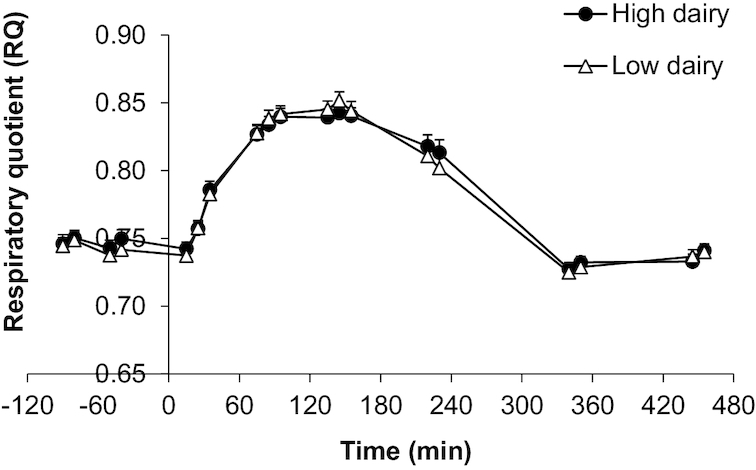
Mean ± SEM RQ after a ^13^C-enriched oral-glucose-tolerance test in overweight men and women (*n* = 45) after a 6-wk high-dairy diet (●) or low-dairy diet (Δ) in a crossover design. Summary measures are presented in Table 5; differences were assessed using linear mixed models. RQ, respiratory quotient.

When comparing the HDD with the LDD, there was no difference in mean RQ curves after the OGTT (**Figure 2**). Fasting RQ, calculated based on 2 RQ measurements before the OGTT, was similar after both diets (LDD: 0.74 ± 0.04; HDD: 0.75 ± 0.04; *P* = 0.560) (**Table 5**). In addition, the fasting values calculated based on the 2 measurements between *t* = 330 and *t* = 480 were the same (LDD: 0.73 ± 0.02 compared with HDD: 0.73 ± 0.02; *P* = 0.879). The mean of the highest RQ value measured between *t* = 60 and *t* = 240 for each individual was 0.87 ± 0.04 for both diets (*P* = 0.954). Consequently, ΔRQ, giving an indication of metabolic flexibility, was the same (LDD: 0.13 ± 0.05; HDD: 0.12 ± 0.04; *P* = 0.707) after both intervention periods. Linear mixed-model analyses indicated no carryover effects. Adjustments of fasting RQ and ΔRQ analyses for body weight, caloric intake, macronutrient intake, fasting and peak insulin concentration, or physical activity did not materially change the results.

**TABLE 5:** Indexes reflecting the metabolic response after a ^13^C-enriched oral-glucose-tolerance test in 45 overweight men and women after a 6-wk HDD or LDD in a crossover design^1^

	Fasting values	Peak values	Time to peak (min)	iAUC (0–2 h)	iAUC (0–4 h)	iAUC (0–8 h)
Glucose, mmol/L						
LDD	5.5 ± 0.3	10.6 ± 1.6	61.0 ± 20.8	397.8 ± 132.7	490.2 ± 176.9	496.3 ± 177.4
HDD	5.5 ± 0.3	10.9 ± 1.7	58.0 ± 21.1	393.6 ± 132.9	484.9 ± 179.3	502.7 ± 184.8
Insulin, µU/mL						
LDD	8.1 ± 2.8	115.9 ± 55.9	69.4 ± 29.5	7621 ± 3700	10,495 ± 5764	10,653 ± 5797
HDD	8.9 ± 3.3*	119.2 ± 59.1	72.2 ± 28.2	7927 ± 4228	11,030 ± 6255	11,135 ± 6304
RQ						
LDD	0.74 ± 0.04	0.87 ± 0.04	147.3 ± 46.5	4.77 ± 3.11	16.96 ± 9.32	21.73 ± 14.75
HDD	0.75 ± 0.04	0.87 ± 0.04	146.4 ± 55.1	4.69 ± 3.11	16.16 ± 6.92	20.56 ± 9.97
RaT, mg · kg^−1^ · min^−1^						
LDD	2.4 ± 0.4	7.5 ± 1.4	43.6 ± 18.2	381.0 ± 94.1	522.1 ± 115.8	523.6 ± 114.3
HDD	2.5 ± 0.3	7.2 ± 1.2	48.8 ± 22.2	366.0 ± 97.1	503.3 ± 113.3	504.2 ± 114.0
RaE, mg · kg^−1^ · min^−1^						
LDD	0.0 ± 0.0	6.5 ± 1.3	49.9 ± 20.4	535.8 ± 99.4	854.8 ± 115.4	971.7 ± 119.9
HDD	0.0 ± 0.0	6.4 ± 1.2	55.5 ± 29.9	534.5 ± 103.0	862.4 ± 116.3	976.0 ± 116.8
EGP, mg · kg^−1^ · min^−1^						
LDD	2.4 ± 0.4	0.32 ± 0.3	119.3 ± 60.9	155.2 ± 35.4	353.1 ± 68.9	618.8 ± 127.6
HDD	2.5 ± 0.3	0.37 ± 0.3	126.6 ± 74.1	169.5 ± 31.6**	377.2 ± 58.2**	671.5 ± 123.7*
GCR, mL · kg^−1^ · min^−1^						
LDD	2.4 ± 0.4	5.1 ± 1.2	120.4 ± 47.0	109.0 ± 81.8	271.4 ± 132.1	284.9 ± 134.5
HDD	2.6 ± 0.4	5.1 ± 1.2	136.1 ± 36.7*	100.0 ± 79.3	261.1 ± 133.1	273.2 ± 134.3
^13^CO_2_ (% dose/h)						
LDD	0.0 ± 0.0	6.8 ± 0.8	233.3 ± 34.4	3.46 ± 0.73	15.01 ± 2.14	34.76 ± 4.38
HDD	0.0 ± 0.0	6.9 ± 0.8	228.0 ± 30.3	3.52 ± 0.73	15.32 ± 1.92	35.44 ± 4.08*

^1^Values are mean ± SD, *n* = 45 (*n* = 43 for glucose kinetics). Differences were assessed using linear mixed models. RQ iAUC is based on deviating time frames (*t* = 0–95, 0–230, and 0–455 min). Because EGP was suppressed after the test meals, the nadir values and time to nadir are presented. Also, the area beneath baseline (decremental AUC) was calculated using mirrored data. For ^13^CO_2_, the cumulative values (% dose) are presented under iAUC. *,**Significantly different from LDD: *P < 0.05, **P < 0.01. EGP, endogenous glucose production; GCR, glucose clearance rate; HDD, high-dairy diet; iAUC, incremental AUC; LDD, low-dairy diet; RaE, rate of appearance of exogenous glucose; RaT, rate of appearance of total glucose; RQ, respiratory quotient.

### Fasting and postprandial glucose and insulin concentrations

**FIGURE 3:**
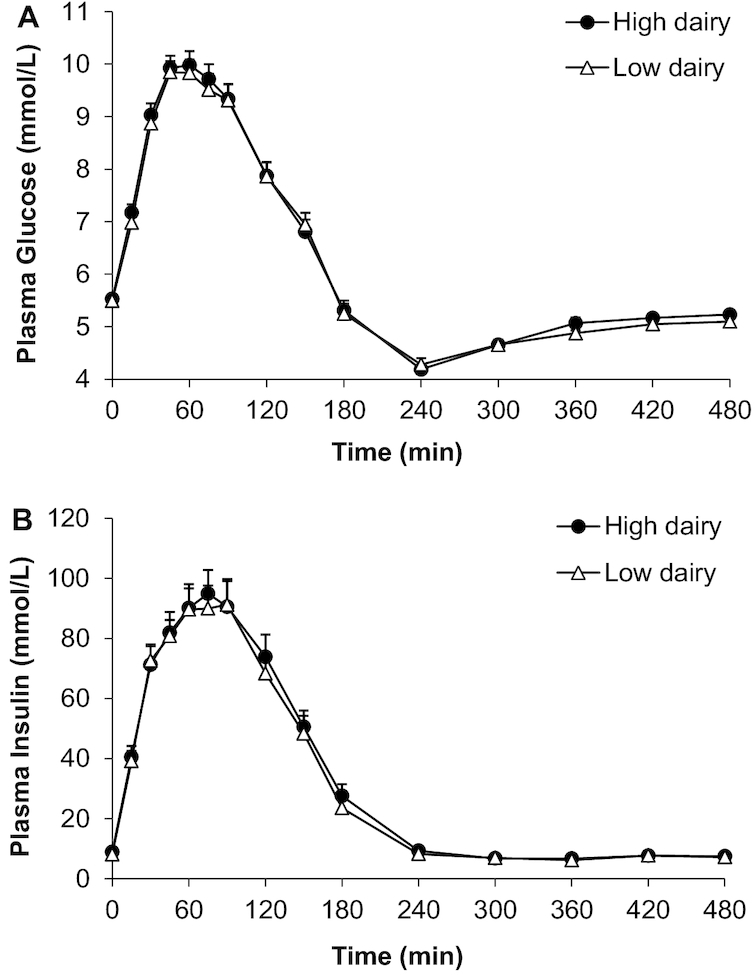
Mean ± SEM concentrations of plasma glucose (A) and plasma insulin (B) after a ^13^C-enriched oral-glucose-tolerance test in overweight men and women (*n* = 45) after a 6-wk high-dairy diet (●) or low-dairy diet (Δ) in a crossover design. Summary measures are presented in Table 5; differences were assessed using linear mixed models.

No differences were found in fasting plasma glucose concentrations, time to peak, peak values, or iAUC after the diets (Table 5). Sixteen subjects appeared to have normal glucose values (both fasting and postprandial), whereas 29 subjects had either impaired fasting glucose (IFG), impaired glucose tolerance (IGT), or both. Based on the glucose values at *t* = 120 there were 22 subjects (49%) with IGT (≥7.8 mmol/L) after one or both dietary periods. On average, glucose concentrations fell below baseline around *t* = 180 and remained below baseline until the end of the test (*t* = 480) (**Figure 3**A).

Fasting insulin concentrations were slightly lower after the LDD (LDD: 8.1 ± 2.8 mU/L; HDD: 8.9 ± 3.3 mU/L; *P* = 0.024). Adjustments of fasting insulin for body weight; fiber intake; and/or intake of coffee, tea, fruit juice, and alcoholic beverages did not materially change the results. Mean postprandial insulin concentrations were the same after both interventions (Figure 3B, Table 5).

### Glucose kinetics

**FIGURE 4:**
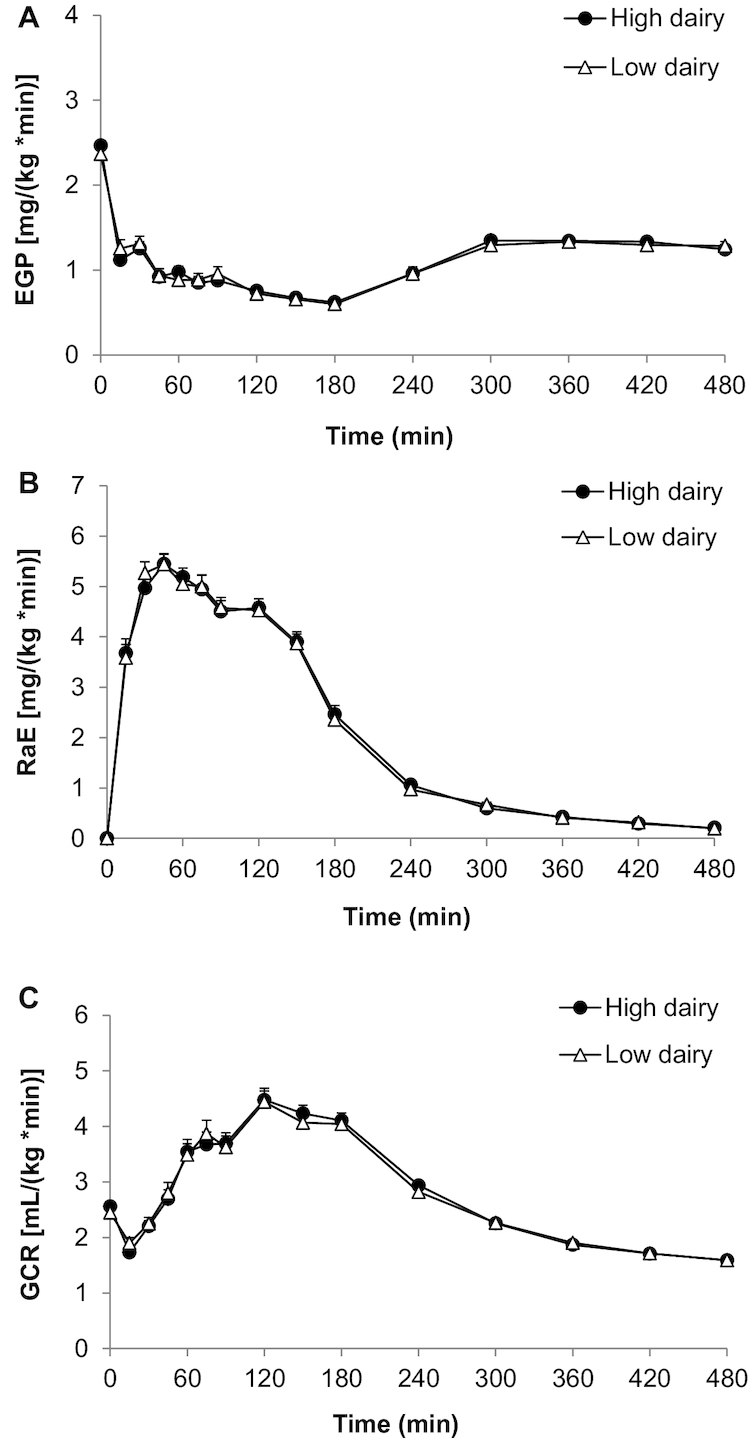
Mean ± SEM EGP (A), RaE (B), and GCR (C) after a ^13^C-enriched oral-glucose-tolerance test in overweight men and women (*n* = 43) after a 6-wk high-dairy diet (●) or low-dairy diet (Δ) in a crossover design. Summary measures are presented in Table 5; differences were assessed using linear mixed models. EGP, endogenous glucose production; GCR, glucose clearance rate; RaE, rate of appearance of exogenous glucose.

No differences were observed in postprandial glucose kinetics. Comparing the response to an OGTT after the HDD and LDD, the mean EGP (**Figure 4**A), RaE (Figure 4B) as well as GCR (Figure 4C) were similar for 8 h after the OGTT. Additional outcomes such as peak value, time to peak, and iAUC are summarized in Table 5. The decremental AUC of EGP was somewhat higher after the HDD, which is likely explained by the “difference” (*P* = 0.051) in baseline value. In addition, a small shift in GCR time to peak was observed (LDD: 120.4 ± 47.0 min compared with HDD: 136.1 ± 36.7 min; *P* = 0.014), but no difference in peak value of GCR, or iAUC representing the total amount of cleared glucose in a specific time period. Linear mixed-model analyses indicated no carryover effects. Adjustments of outcomes for body weight did not materially change the results.

### ^13^CO_2_ excretion in breath

**FIGURE 5:**
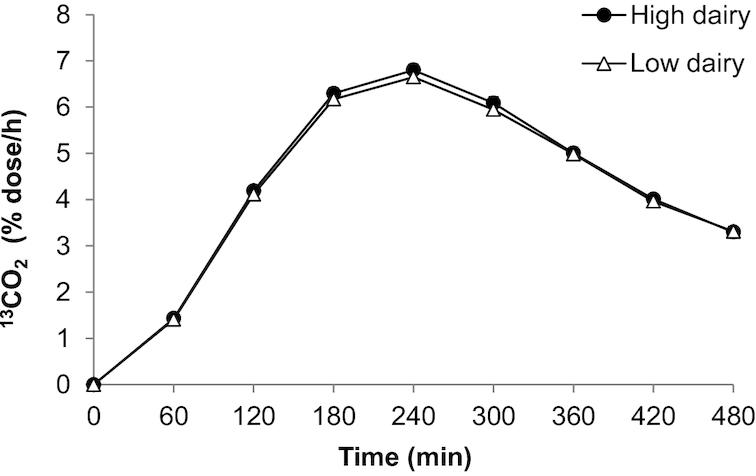
Mean ± SEM ^13^CO_2_ in breath after a ^13^C-enriched oral-glucose-tolerance test in overweight men and women (*n* = 45) after a 6-wk high-dairy diet (●) or low-dairy diet (Δ) in a crossover design. Summary measures are presented in Table 5; differences were assessed using linear mixed models.

^13^CO_2_ excretion in breath, reflecting the rate of oxidation of the ^13^C-labeled substrate (OGTT), was similar after both diets (**Figure 5**, Table 5).

### Insulin sensitivity

Due to the difference in fasting insulin values, HOMA-IR was higher after HDD (LDD: 1.99 ± 0.72 compared with HDD: 2.21 ± 0.91; *P* = 0.027) and HOMA-β showed a similar trend (LDD: 82.1 ± 28.8 compared with HDD: 87.2 ± 28.5; *P* = 0.076).

The Matsuda index, representing a composite of both hepatic and peripheral tissue IS, did not differ significantly (LDD: 4.12 ± 1.75 compared with HDD: 3.84 ± 1.47; *P* = 0.077), but suggested a similar trend. In addition, IS calculated based on GCR (iAUCs) and mean postprandial insulin concentrations were the same after both dairy interventions (data not shown). Adjustments of IS-related outcomes for body weight; fiber intake; and/or intake of coffee, tea, fruit juice, and alcoholic beverages did not materially change the results.

### Inflammation and oxidative stress

Median high-sensitivity C-reactive protein, as a marker of systemic inflammation, did not differ statistically between both diets (LDD: 1.00 [0.60–2.00] mg/L compared with HDD: 1.20 [0.80–2.55] mg/L; *P* = 0.065). The oxidative stress marker MDA was not different after both diets based on plasma values (LDD: 0.51 [0.45–0.65] µmol/L compared with HDD: 0.48 [0.43–0.59] µmol/L; *P* = 0.196), although 24-h urinary excretion was higher after HDD (LDD: 3.04 [2.55–4.12] µmol/24 h compared with HDD: 4.38 [3.18–6.56] µmol/24 h; *P* = 0.001). The 24-h urinary excretion of the oxidative stress marker 8-OHdG was similar after both dietary interventions (LDD: 408.9 [353.1–478.7] µg/24 h compared with HDD: 400.0 [372.3–478.5] µg/24 h; *P* = 0.745) (Table 4).

### Appetite and feeling of discomfort

During the test days, no difference in appetite was observed in the hours after the OGTT (data not shown), nor in complaints such as headache, nausea, tiredness, sweating, and trembling when comparing the HDD and LDD.

### Subgroup analyses

As secondary post hoc analyses we assessed for 14 outcomes (see the Methods section for details) whether there were any different outcomes between metabolically different subgroups (based on BMI, fasting glucose, and HOMA-IR). We found a significant interaction (fasting glucose × diet) for glucose iAUC 0–4 h (*P* = 0.050), indicating a different effect of the dairy diets on the glucose response between subjects with IFG and those with NFG. After the HDD the 0–4 h iAUC of glucose was decreased in the NFG group (441.0 ± 160.6) and increased in the IFG group (530.9 ± 190.0) compared with the 0–4 h iAUC of glucose after the LDD (NFG: 490.0 ± 168.6 and IFG: 490.3 ± 189.3). Further, we found a significant interaction (HOMA-IR × diet) for GCR iAUC 0–4 h (*P* = 0.008), suggesting an opposite effect in glucose clearance between subjects with a low and those with a high HOMA-IR index (less or more than 2.56, before intervention). After the HDD the GCR (0–4 h iAUC) was increased in the low HOMA-IR group (293.6 ± 123.5) and decreased in the high HOMA-IR group (223.8 ± 136.9) compared with the GCR (0–4 h iAUC) after the LDD (low HOMA-IR: 262.1 ± 127.9 and high HOMA-IR: 268.2 ± 151.8).

## Discussion

Dairy intake is often associated with improvements in cardiometabolic health. In this randomized crossover study we investigated the effects of a 6-wk HDD and LDD on parameters of metabolic flexibility and postprandial glucose kinetics after a 75-g ^13^C-enriched OGTT in middle-aged overweight men and women. No differences were observed in these postprandial outcomes between both dietary interventions.

To our knowledge, the effect of dairy intake on postprandial metabolic flexibility per se has not yet been studied. In the present study, we assessed metabolic flexibility using an OGTT after 6 wk of HDD or LDD. Because the flexibility of skeletal muscle to switch between oxidizing fat and glucose is also related to IS, glycogen stores, percentage body fat, and fitness (20), factors like bodyweight and physical activity, as well as the evening meal before the OGTT, were kept as stable as possible during both dietary periods. Although instructed by dieticians to substitute in order to have a weight-maintenance diet, the reported difference between the HDD and the LDD was ∼100 kcal/d, which is comparable with 1 dairy portion, and in agreement with higher protein intake during the HDD. The current trial was done in low–medium dairy consumers, who would have to leave out several, often habitual, foods during the HDD in order to consume an isocaloric diet. The difficulty of changing a habit in eating behavior (44) is in agreement with the experiences of several subjects during the HDD, who indicated that to reach the goal of 5–6 dairy portions, an “extra” portion had to be consumed before bedtime. This may explain the small difference (∼0.4 kg) in mean body weight, consistent with a trend towards a higher fat mass after the HDD (∼0.5 kg). A similar weight gain was observed by Benatar et al. (45) in the group that increased dairy intake for 1 mo. Although weight loss is known to improve IS, the small difference in weight observed in the present study seems unlikely to have influenced the results, as confirmed by the secondary analyses performed taking body weight into account. In addition, muscle mass and physical activity, which also affect IS and metabolic flexibility, remained stable.

In general, positive findings of dairy intake related to metabolic health may be partly due to the associations of some single nutrients in dairy products, such as magnesium and calcium, with IS (9). Intake of these micronutrients was higher during the HDD, particularly calcium intake, which more than doubled during the HDD. In addition, the type of dairy (both product type and fat content) may also be of importance. Low-fat dairy is often associated with beneficial cardiometabolic effects, but there are also indications that full-fat dairy has positive effects (7, 11), despite the higher content of SFAs. Full-fat dairy is mainly linked to IS via fatty acids such as *trans*-palmitoleic acid (11, 46) and it may thus be attributed to specific components in the dairy fat. Unfortunately, nutritional data from the present study did not differentiate between TFA intake of industrial or ruminant origin, although their cardiometabolic effects may be different. However, although we observed no difference in total TFA intake, it may be assumed that during HDD a larger part of TFA was of ruminant origin (mainly from cheese), whereas during the LDD a larger part of TFA was of industrial origin. Other studies suggest that especially fermented dairy, and yoghurt in particular, shows the strongest associations with metabolic health (8, 47, 48). Therefore, ≥2 portions of yoghurt had to be consumed during the HDD, as well as 1–2 portions of cheese. Nevertheless, our study could not provide indications or confirmation of such effects.

We did observe a slight difference in fasting insulin and thereby HOMA-IR between the diets. Similar results were found in a crossover study in overweight and obese individuals by Turner et al. (49), where a 4-wk high-dairy diet (4–6 dairy portions) increased fasting insulin and HOMA-IR compared with high red meat intake and a control diet. In contrast, in a 12-wk study in overweight and obese subjects an adequate dairy diet (>3.5 servings/d) improved IS (50). Interestingly, this effect on HOMA-IR was already visible after 1 wk, and was less pronounced and consistent after 4 wk. A 6-mo trial in healthy subjects consuming 4 servings of low-fat dairy per day also improved HOMA-IR (−11%) owing to reduced fasting insulin concentrations compared with intake of <2 servings/d (15). Another 6-mo intervention also found a difference in HOMA-IR between the “milk group” (3–5 portions of dairy products) and the control group (habitual diet), but this was due to an unexpected increase in insulin and HOMA-IR in the control group, whereas the “milk group” values remained stable (51). Several other studies observed a neutral effect of dairy on fasting glucose and insulin concentrations (45, 52, 53). A recent review emphasized the mixed nature of results from interventions on IS measures, even when comparing the effects of weight-stable dairy intervention trials (12). Interventions that study the effects of dairy on whole-body IS with an additional OGTT (using Matsuda index), thereby going beyond measures of mainly hepatic IS, are rather scarce. The study by Turner et al. (49) found a decrease in IS (Matsuda) after 4 wk of high dairy, whereas a 6-mo RCT in overweight and obese subjects comparing milk intake (1 L/d) with other beverages found no effect (53).

We aimed for our 6-wk intervention period to be sufficiently long to induce an effect on postprandial glucose metabolism and IS and not too long with respect to compliance of the subjects to the dietary obligations and restrictions. Although effects on HOMA-IR have been observed after shorter interventions (49, 50), this period may not be long enough to affect postprandial glucose metabolism or metabolic flexibility. Another possible reason for a lack of effect may be related to the amount of dairy. The large difference in dairy intake between both interventions we aimed for was based on extremes in the amount of dairy intake in the Netherlands [≤1 compared with 5 (women) or 6 (men) portions per day]. It is however possible that for the general population the optimal amount of dairy intake is more moderate or that the requirements differ for different individuals or subgroups, as the results of our post hoc subgroup analyses may suggest. Interestingly, the effect of dairy (especially whey protein) on glucose metabolism and T2DM risk may depend on the person's specific glucokinase gene polymorphism which is related to insulin resistance (54), indicating that some persons may benefit more from high dairy intake than others.

The subjects in the selected age- and BMI-range were expected to have an increased risk of the development of diabetes, but around 35% appeared to have normal glucose values (both fasting and postprandial), and 49% was IGT during ≥1 of the OGTTs. Possibly, an effect of dairy intake on postprandial glucose metabolism could be expected in subjects with a higher BMI or age range.

Because of the large difference in dairy intake and the aim of weight maintenance, the intake of other food products during the LDD and HDD differed substantially. During the LDD there was a higher intake of beverages such as coffee, tea, and fruit juice as well as increased consumption of bread and bread toppings such as meat products. During the HDD the intake of breakfast cereals was increased, likely combined with the milk or yoghurt portion. Despite this increase, dietary fiber intake was higher during the LDD. It should be noted that these products (or their ingredients) may also influence IS or glucose metabolism. Whole grain and fiber intake are associated with improved IS and a lower risk of T2DM (55). Coffee (56), tea (57, 58), and moderate alcohol (59, 60) consumption have also been associated with a reduced risk of diabetes, which may suggest an influence of these beverages on IS. Habitual intake of sugar-sweetened beverages and juices is mainly associated with a greater incidence of diabetes (61), although a recent meta-analysis of RCTs suggested a neutral effect of 100% fruit juice on glycemic control (62). Adjustment for differences in intake of fiber, coffee, tea, fruit juice, and alcoholic beverages did not materially affect the results of our study.

A strength of this study was the large difference in prescribed dairy intake (≥4 portions/d) between the 2 intervention periods. Furthermore, by making use of a sophisticated method using stable isotopes to thoroughly study glucose kinetics, possible changes in postprandial glucose metabolism underlying the total glucose response were ruled out. The free-living situation, where the participants could choose their own substitution diet and had some freedom regarding the choice of milk and buttermilk, had both advantages and disadvantages. It created a situation which was closest to their habitual situation, increasing external validity, but it made the dietary intervention less controlled. No conclusions can be drawn relating a particular type of dairy to our outcomes, as the focus was on the amount of dairy. In addition, the use of buttermilk and reduced-fat cheese may not be common in other countries, possibly limiting comparability with other studies.

To conclude, in this randomized controlled crossover intervention trial, the amount of dairy intake (high compared with low) during a 6-wk period had a neutral effect on metabolic flexibility and postprandial glucose metabolism in middle-aged overweight subjects. More intervention trials are needed to study the effects of dairy intake on metabolic health, possibly making a distinction between subjects’ metabolic subgroups or differentiating between specific types of dairy products or dairy fat content, notably full-fat compared with low-fat dairy.

## Supplementary Material

nqz017_Supplemental_Files

## Notes

Supported by Public-Private Partnership—TKI Agro and Food grant TKI-AF-12104; the University Medical Center Groningen (UMCG) and FrieslandCampina (FC) participated in this partnership.

Researchers from both FC and UMCG were involved in the design of the study, the study was conducted at the UMCG, and data analyses and interpretation were performed by researchers from the UMCG.

Supplemental Table 1 and Supplemental Figure 1 are available from the “Supplementary data” link in the online posting of the article and from the same link in the online table of contents at https://academic.oup.com/ajcn/.

Abbreviations used: EGP, endogenous glucose production; GC-MS, gas chromatography–mass spectrometry; GCR, glucose clearance rate; HDD, high-dairy diet; iAUC, incremental AUC; IFG, impaired fasting glucose; IGT, impaired glucose tolerance; IS, insulin sensitivity; LDD, low-dairy diet; MDA, malondialdehyde; NFG, normal fasting glucose; OGTT, oral-glucose-tolerance test; RaE, rate of appearance of exogenous glucose; RaT, rate of appearance of total glucose; RQ, respiratory quotient; *t*, time; TFA, *trans* fatty acid; T2DM, type 2 diabetes mellitus.

## References

[1] Pereira MA, Jacobs DR Jr, Van Horn L, Slattery ML, Kartashov AI, Ludwig DS Dairy consumption, obesity, and the insulin resistance syndrome in young adults: the CARDIA study. JAMA. 2002;287:2081–9. 11966382 10.1001/jama.287.16.2081

[2] Azadbakht L, Mirmiran P, Esmaillzadeh A, Azizi F Dairy consumption is inversely associated with the prevalence of the metabolic syndrome in Tehranian adults. Am J Clin Nutr. 2005;82:523–30. 16155263 10.1093/ajcn.82.3.523

[3] Ruidavets JB, Bongard V, Dallongeville J, Arveiler D, Ducimetiere P, Perret B, Simon C, Amouyel P, Ferrieres J High consumptions of grain, fish, dairy products and combinations of these are associated with a low prevalence of metabolic syndrome. J Epidemiol Community Health. 2007;61:810–17. 17699537 10.1136/jech.2006.052126 PMC2660006

[4] Aune D, Norat T, Romundstad P, Vatten LJ Dairy products and the risk of type 2 diabetes: a systematic review and dose-response meta-analysis of cohort studies. Am J Clin Nutr. 2013;98:1066–83. 23945722 10.3945/ajcn.113.059030

[5] Gao D, Ning N, Wang C, Wang Y, Li Q, Meng Z, Liu Y, Li Q Dairy products consumption and risk of type 2 diabetes: systematic review and dose-response meta-analysis. PLoS One. 2013;8:e73965. 24086304 10.1371/journal.pone.0073965 PMC3785489

[6] Hirahatake KM, Slavin JL, Maki KC, Adams SH Associations between dairy foods, diabetes, and metabolic health: potential mechanisms and future directions. Metabolism. 2014;63:618–27. 24636056 10.1016/j.metabol.2014.02.009 PMC5367265

[7] Drouin-Chartier J, Brassard D, Tessier-Grenier M, Côté JA, Labonté M, Desroches S, Couture P, Lamarche B Systematic review of the association between dairy product consumption and risk of cardiovascular-related clinical outcomes. Adv Nutr. 2016;7:1026–40. 28140321 10.3945/an.115.011403 PMC5105032

[8] Gijsbers L, Ding EL, Malik VS, de Goede J, Geleijnse JM, Soedamah-Muthu SS Consumption of dairy foods and diabetes incidence: a dose-response meta-analysis of observational studies. Am J Clin Nutr. 2016;103:1111–24. 26912494 10.3945/ajcn.115.123216

[9] Ma B, Lawson AB, Liese AD, Bell RA, Mayer-Davis EJ Dairy, magnesium, and calcium intake in relation to insulin sensitivity: approaches to modeling a dose-dependent association. Am J Epidemiol. 2006;164:449–58. 16861328 10.1093/aje/kwj246

[10] Chartrand D, Da Silva MS, Julien P, Rudkowska I Influence of amino acids in dairy products on glucose homeostasis: the clinical evidence. Can J Diabetes. 2017;41:329–37. 28233627 10.1016/j.jcjd.2016.10.009

[11] Nestel PJ, Straznicky N, Mellett NA, Wong G, De Souza DP, Tull DL, Barlow CK, Grima MT, Meikle PJ Specific plasma lipid classes and phospholipid fatty acids indicative of dairy food consumption associate with insulin sensitivity. Am J Clin Nutr. 2013;99:46–53. 24153346 10.3945/ajcn.113.071712

[12] Turner KM, Keogh JB, Clifton PM Dairy consumption and insulin sensitivity: a systematic review of short- and long-term intervention studies. Nutr Metab Cardiovasc Dis. 2015;25:3–8. 25156891 10.1016/j.numecd.2014.07.013

[13] Crichton GE, Howe PR, Buckley JD, Coates AM, Murphy KJ Dairy consumption and cardiometabolic health: outcomes of a 12-month crossover trial. Nutr Metab (Lond). 2012;9:19. 22433747 10.1186/1743-7075-9-19 PMC3348063

[14] Benatar JR, Sidhu K, Stewart RA Effects of high and low fat dairy food on cardio-metabolic risk factors: a meta-analysis of randomized studies. PloS One. 2013;8:e76480. 24146877 10.1371/journal.pone.0076480 PMC3795726

[15] Rideout TC, Marinangeli CP, Martin H, Browne RW, Rempel CB Consumption of low-fat dairy foods for 6 months improves insulin resistance without adversely affecting lipids or bodyweight in healthy adults: a randomized free-living cross-over study. Nutr J. 2013;12:56. 23638799 10.1186/1475-2891-12-56 PMC3651862

[16] van Ommen B, Keijer J, Heil SG, Kaput J Challenging homeostasis to define biomarkers for nutrition related health. Mol Nutr Food Res. 2009;53:795–804. 19517455 10.1002/mnfr.200800390

[17] Huber M, Knottnerus JA, Green L, van der Horst H, Jadad AR, Kromhout D, Leonard B, Lorig K, Loureiro MI, van der Meer JWM et al. How should we define health?. BMJ. 2011;343:d4163. 21791490 10.1136/bmj.d4163

[18] Wopereis S, Wolvers D, van Erk M, Gribnau M, Kremer B, van Dorsten FA, Boelsma E, Garczarek U, Cnubben N, Frenken L et al. Assessment of inflammatory resilience in healthy subjects using dietary lipid and glucose challenges. BMC Med Genomics. 2013;6:44. 24160467 10.1186/1755-8794-6-44 PMC4015956

[19] Galgani JE, Moro C, Ravussin E Metabolic flexibility and insulin resistance. Am J Physiol Endocrinol Metab. 2008;295:E1009–17. 18765680 10.1152/ajpendo.90558.2008 PMC2584808

[20] Kelley DE Skeletal muscle fat oxidation: timing and flexibility are everything. J Clin Invest. 2005;115:1699–702. 16007246 10.1172/JCI25758 PMC1159159

[21] Storlien L, Oakes ND, Kelley DE Metabolic flexibility. Proc Nutr Soc. 2004;63:363–8. 15294056 10.1079/PNS2004349

[22] van Hooijdonk T, Hettinga K Dairy in a sustainable diet: a question of balance. Nutr Rev. 2015;73:48–54. 26175490 10.1093/nutrit/nuv040

[23] Drewnowski A Measures and metrics of sustainable diets with a focus on milk, yogurt, and dairy products. Nutr Rev. 2018;76:21–8. 29206982 10.1093/nutrit/nux063 PMC5914342

[24] Smith RL, Soeters MR, Wüst RC, Houtkooper RH Metabolic flexibility as an adaptation to energy resources and requirements in health and disease. Endocr Rev. 2018;39:489–517. 29697773 10.1210/er.2017-00211 PMC6093334

[25] Fenech MF Dietary reference values of individual micronutrients and nutriomes for genome damage prevention: current status and a road map to the future. Am J Clin Nutr. 2010;91:1438S–54S. 20219957 10.3945/ajcn.2010.28674D

[26] Ashworth CJ, Antipatis C Micronutrient programming of development throughout gestation. Reproduction. 2001;122:527–35. 11570959

[27] Melanson EL, Donahoo WT, Dong F, Ida T, Zemel MB Effect of low- and high-calcium dairy-based diets on macronutrient oxidation in humans. Obes Res. 2005;13:2102–12. 16421344 10.1038/oby.2005.261

[28] Cederholm J, Wibell L Insulin release and peripheral sensitivity at the oral glucose tolerance test. Diabetes Res Clin Pract. 1990;10:167–75. 2261853 10.1016/0168-8227(90)90040-z

[29] Priebe MG, Wang H, Weening D, Schepers M, Preston T, Vonk RJ Factors related to colonic fermentation of nondigestible carbohydrates of a previous evening meal increase tissue glucose uptake and moderate glucose-associated inflammation. Am J Clin Nutr. 2010;91:90–7. 19889821 10.3945/ajcn.2009.28521

[30] American Diabetes Association. Diagnosis and classification of diabetes mellitus. Diabetes Care. 2014;37(Suppl 1):S81–90. 24357215 10.2337/dc14-S081

[31] Altman DG, Bland JM Treatment allocation by minimisation. BMJ. 2005;330:843. 15817555 10.1136/bmj.330.7495.843 PMC556084

[32] Van Rossum CTM, Fransen HP, Verkaik-Kloosterman J, Buurma-Rethans EJM, Ocké MC Dutch National Food Consumption Survey 2007–2010: diet of children and adults aged 7 to 69 years. Bilthoven: National Institute for Public Health and the Environment; 2011.

[33] Baecke JA, Burema J, Frijters JE A short questionnaire for the measurement of habitual physical activity in epidemiological studies. Am J Clin Nutr. 1982;36:936–42. 7137077 10.1093/ajcn/36.5.936

[34] Heymsfield SB, Arteaga C, McManus C, Smith J, Moffitt S Measurement of muscle mass in humans: validity of the 24-hour urinary creatinine method. Am J Clin Nutr. 1983;37:478–94. 6829490 10.1093/ajcn/37.3.478

[35] Hanff E, Eisenga MF, Beckmann B, Bakker SJ, Tsikas D Simultaneous pentafluorobenzyl derivatization and GC-ECNICI-MS measurement of nitrite and malondialdehyde in human urine: close positive correlation between these disparate oxidative stress biomarkers. J Chromatogr B Analyt Technol Biomed Life Sci. 2017;1043:167–75. 10.1016/j.jchromb.2016.07.027 27461359

[36] Boers HM, van Dijk TH, Hiemstra H, Hoogenraad A, Mela DJ, Peters HP, Vonk RJ, Priebe MG Effect of fibre additions to flatbread flour mixes on glucose kinetics: a randomised controlled trial. Br J Nutr. 2017;118:777–87. 29110741 10.1017/S0007114517002781

[37] Eelderink C, Schepers M, Preston T, Vonk RJ, Oudhuis L, Priebe MG Slowly and rapidly digestible starchy foods can elicit a similar glycemic response because of differential tissue glucose uptake in healthy men. Am J Clin Nutr. 2012;96:1017–24. 22990033 10.3945/ajcn.112.041947

[38] Steele R, Wall JS, De Bodo RC, Altszuler N Measurement of size and turnover rate of body glucose pool by the isotope dilution method. Am J Physiol. 1956;187:15–24. 13362583 10.1152/ajplegacy.1956.187.1.15

[39] De Bodo RC, Steele R, Altszuler N, Dunn A, Bishop JS On the hormonal regulation of carbohydrate metabolism; studies with C14 glucose. Recent Prog Horm Res. 1963;19:445–88. 14284029

[40] Tissot S, Normand S, Guilluy R, Pachiaudi C, Beylot M, Laville M, Cohen R, Mornex R, Riou JP Use of a new gas chromatograph isotope ratio mass spectrometer to trace exogenous ^13^C labelled glucose at a very low level of enrichment in man. Diabetologia. 1990;33:449–56. 2210116 10.1007/BF00405104

[41] Schenk S, Davidson CJ, Zderic TW, Byerley LO, Coyle EF Different glycemic indexes of breakfast cereals are not due to glucose entry into blood but to glucose removal by tissue. Am J Clin Nutr. 2003;78:742–8. 14522732 10.1093/ajcn/78.4.742

[42] Matthews D, Hosker J, Rudenski A, Naylor B, Treacher D, Turner R Homeostasis model assessment: insulin resistance and β-cell function from fasting plasma glucose and insulin concentrations in man. Diabetologia. 1985;28:412–19. 3899825 10.1007/BF00280883

[43] Matsuda M, DeFronzo RA Insulin sensitivity indices obtained from oral glucose tolerance testing: comparison with the euglycemic insulin clamp. Diabetes Care. 1999;22:1462–70. 10480510 10.2337/diacare.22.9.1462

[44] van't Riet J, Sijtsema SJ, Dagevos H, De Bruijn G The importance of habits in eating behaviour. An overview and recommendations for future research. Appetite. 2011;57:585–96. 21816186 10.1016/j.appet.2011.07.010

[45] Benatar JR, Jones E, White H, Stewart RA A randomized trial evaluating the effects of change in dairy food consumption on cardio-metabolic risk factors. Eur J Prev Cardiol. 2014;21:1376–86. 23774272 10.1177/2047487313493567

[46] Kratz M, Marcovina S, Nelson JE, Yeh MM, Kowdley KV, Callahan HS, Song X, Di C, Utzschneider KM Dairy fat intake is associated with glucose tolerance, hepatic and systemic insulin sensitivity, and liver fat but not β-cell function in humans. Am J Clin Nutr. 2014;99:1385–96. 24740208 10.3945/ajcn.113.075457 PMC4021783

[47] O'Connor LM, Lentjes MA, Luben RN, Khaw K, Wareham NJ, Forouhi NG Dietary dairy product intake and incident type 2 diabetes: a prospective study using dietary data from a 7-day food diary. Diabetologia. 2014;57:909–17. 24510203 10.1007/s00125-014-3176-1 PMC3980034

[48] Chen M, Sun Q, Giovannucci E, Mozaffarian D, Manson JE, Willett WC, Hu FB Dairy consumption and risk of type 2 diabetes: 3 cohorts of US adults and an updated meta-analysis. BMC Medicine. 2014;12:215. 25420418 10.1186/s12916-014-0215-1 PMC4243376

[49] Turner KM, Keogh JB, Clifton PM Red meat, dairy, and insulin sensitivity: a randomized crossover intervention study. Am J Clin Nutr. 2015;101:1173–9. 25809854 10.3945/ajcn.114.104976

[50] Stancliffe RA, Thorpe T, Zemel MB Dairy attentuates oxidative and inflammatory stress in metabolic syndrome. Am J Clin Nutr. 2011;94:422–30. 21715516 10.3945/ajcn.111.013342 PMC3142721

[51] Wennersberg MH, Smedman A, Turpeinen AM, Retterstøl K, Tengblad S, Lipre E, Aro A, Mutanen P, Seljeflot I, Basu S Dairy products and metabolic effects in overweight men and women: results from a 6-mo intervention study. Am J Clin Nutr. 2009;90:960–8. 19710195 10.3945/ajcn.2009.27664

[52] van Meijl LE, Mensink RP Low-fat dairy consumption reduces systolic blood pressure, but does not improve other metabolic risk parameters in overweight and obese subjects. Nutr Metab Cardiovasc Dis. 2011;21:355–61. 20153619 10.1016/j.numecd.2009.10.008

[53] Engel S, Tholstrup T, Bruun JM, Astrup A, Richelsen B, Raben A Effect of high milk and sugar-sweetened and non-caloric soft drink intake on insulin sensitivity after 6 months in overweight and obese adults: a randomized controlled trial. Eur J Clin Nutr. 2017;72(3):358–66. 29235560 10.1038/s41430-017-0006-9

[54] Da Silva MS, Chartrand D, Vohl M, Barbier O, Rudkowska I Dairy product consumption interacts with glucokinase (GCK) gene polymorphisms associated with insulin resistance. J Pers Med. 2017;7:8. 10.3390/jpm7030008 PMC5618154 28867816

[55] Weickert MO, Pfeiffer AF Impact of dietary fiber consumption on insulin resistance and the prevention of type 2 diabetes. J Nutr. 2018;148:7–12. 29378044 10.1093/jn/nxx008

[56] Huxley R, Lee CMY, Barzi F, Timmermeister L, Czernichow S, Perkovic V, Grobbee DE, Batty D, Woodward M Coffee, decaffeinated coffee, and tea consumption in relation to incident type 2 diabetes mellitus: a systematic review with meta-analysis. Arch Intern Med. 2009;169:2053–63. 20008687 10.1001/archinternmed.2009.439

[57] Stote KS, Baer DJ Tea consumption may improve biomarkers of insulin sensitivity and risk factors for diabetes. J Nutr. 2008;138:1584S–8S. 18641211 10.1093/jn/138.8.1584S

[58] Yang W, Wang W, Fan W, Deng Q, Wang X Tea consumption and risk of type 2 diabetes: a dose-response meta-analysis of cohort studies. Br J Nutr. 2014;111:1329–39. 24331002 10.1017/S0007114513003887

[59] Schrieks IC, Heil AL, Hendriks HF, Mukamal KJ, Beulens JW The effect of alcohol consumption on insulin sensitivity and glycemic status: a systematic review and meta-analysis of intervention studies. Diabetes Care. 2015;38:723–32. 25805864 10.2337/dc14-1556

[60] Li X, Yu F, Zhou Y, He J Association between alcohol consumption and the risk of incident type 2 diabetes: a systematic review and dose-response meta-analysis. Am J Clin Nutr. 2016;103:818–29. 26843157 10.3945/ajcn.115.114389

[61] Imamura F, O'Connor L, Ye Z, Mursu J, Hayashino Y, Bhupathiraju SN, Forouhi NG Consumption of sugar sweetened beverages, artificially sweetened beverages, and fruit juice and incidence of type 2 diabetes: systematic review, meta-analysis, and estimation of population attributable fraction. BMJ. 2015;351:h3576. 26199070 10.1136/bmj.h3576 PMC4510779

[62] Murphy MM, Barrett EC, Bresnahan KA, Barraj LM 100 % Fruit juice and measures of glucose control and insulin sensitivity: a systematic review and meta-analysis of randomised controlled trials. J Nutr Sci. 2017;6:e59. 29299307 10.1017/jns.2017.63 PMC5736636

