# Supplementary material for: The effect of high compared with low dairy consumption on glucose metabolism, insulin sensitivity, and metabolic flexibility in overweight adults: a randomized crossover trial
Source: Am J Clin Nutr. 2019 Apr 17;109(6):1555–68. doi: 10.1093/ajcn/nqz017 (PMC6537937; doi:10.1093/ajcn/nqz017)
Supplement: nqz017_Supplemental_Files [file nqz017_supplemental_files.zip › OSM-Supplemental table 1.docx]

**Supplemental table 1.** Intake of product groups during a 6-week LDD or HDD diet^1^

| Product group (g/day) | **Low dairy** | **High dairy** | P-value |
| --- | --- | --- | --- |
| Coffee | 550.8 [370.8-693.8] | 459.2 [331.3-654.2] | 0.003 |
| Tea | 233.3 [50.0-565.0] | 166.7 [0.0-491.7] | 0.007 |
| Sugar-sweetened beverages | 0.0 [0.0-45.8] | 0.0 [0.0-25.0] | 0.054 |
| Artificially sweetened beverages | 0.0 [0.0-25.0] | 0.0 [0.0-25.0] | 0.554 |
| Lemonade | 0.0 [0.0-2.9] | 0.0 [0.0-5.6] | 0.577 |
| Fruit juice | 50.2 [1.7-144.3] | 33.3 [0.0-89.2] | 0.001 |
| Alcoholic beverage, non-sweetened | 160.0 [59.2-353.8] | 125.0 [37.9-312.5] | <0.001 |
| Alcoholic beverage, sweetened | 0.0 [0.0-26.7] | 0.0 [0.0-18.3] | 0.476 |
| Water | 175.0 [97.0-312.5] | 116.7 [1.8-325.3] | 0.182 |
| Bread, whole grain | 29.2 [0.0-59.2] | 11.7 [0.0-53.3] | 0.137 |
| Bread, brown | 46.7 [7.5-97.1] | 35.8 [9.2-78.8] | 0.569 |
| Bread, white | 35.0 [18.3-61.3] | 32.5 [13.5-56.3] | 0.223 |
| Breakfast cereal, unsweetened | 0.0 [0.0-4.6] | 2.1 [0.0-18.3] | 0.001 |
| Breakfast cereal, sweetened | 0.0 [0.0-0.0] | 0.0 [0.0-11.3] | 0.001 |
| Bread topping, savoury | 0.0 [0.0-12.1] | 0.0 [0.0-7.9] | 0.629 |
| Bread topping, sweat | 14.2 [5.0-25.4] | 6.7 [0.0-18.3] | 0.006 |
| Bread topping, meat | 23.3 [7.9-38.8] | 7.8 [0.0-23.8] | 0.001 |
| Peanut butter | 0.0 [0.0-3.8] | 0.0 [0.0-2.5] | 0.079 |
| Nuts, seeds & peanut, unprocessed | 2.5 [0.0-15.0] | 0.0 [0.0-8.6] | 0.027 |
| Nuts, seeds & peanut, processed | 0.0 [0.0-11.3] | 0.0 [0.0-7.5] | 0.277 |
| Pastry and biscuits | 40.0 [18.1-72.9] | 37.5 [16.7-60.5] | 0.037 |
| Eggs | 16.7 [4.9-33.3] | 12.5 [0.0-25.0] | 0.194 |
| Fruits | 127.5 [40.0-190.6] | 87.1 [26.0-209.0] | 0.797 |
| Vegetables, natural | 133.2 [90.8-154.8] | 125.2 [106.3-167.6] | 0.524 |
| Vegetables, prepared/processed | 4.2 [0.0-22.5] | 6.7 [0.0-25.8] | 0.671 |
| Legumes | 0.0 [0.0-33.3] | 0.0 [0.0-25.0] | 0.168 |
| Rice, pasta, grain, brown/whole grain | 0.0 [0.0-0.0] | 0.0 [0.0-0.0] | 0.289 |
| Rice, pasta, grain, white | 30.0 [11.3-44.6] | 22.5 [6.7-33.3] | 0.044 |
| Potato, natural/unprocessed | 45.8 [15.8-74.9] | 30.0 [16.7-66.7] | 0.285 |
| Potato, prepared/processed | 0.0 [0.0-35.0] | 25.0 [0.0-41.7] | 0.414 |
| Savoury dishes, ready to eat | 13.3 [0.0-80.0] | 17.5 [0.0-58.8] | 0.551 |
| Savoury snacks | 6.2 [0.0-20.0] | 6.7 [0.0-18.6] | 0.166 |
| Savoury sauces | 10.8 [5.8-26.3] | 8.4 [3.3-17.1] | 0.115 |
| Cheese 30+ | 10.0 [2.5-15.0] | 55.0 [40.0-60.0] | <0.001 |
| Milk, semi-skimmed | 0.0 [0.0-62.9] | 250.0 [3.3-360.7] | <0.001 |
| Yoghurt, semi-skimmed | 50.0 [0.0-100.0] | 400.0 [366.7-433.3] | <0.001 |
| Buttermilk | 0.0 [0.0-22.9] | 208.3 [0.0-250.0] | <0.001 |
| Other dairy | 1.3 [0.0-20.8] | 0.0 [0.0-14.5] | 0.001 |
| Meat, unprocessed (<5 gram SF) | 0.0 [0.0-24.2] | 0.0 [0.0-23.1] | 0.866 |
| Meat, unprocessed (>5 gram SF) | 0.0 [0.0-0.0] | 0.0 [0.0-16.7] | 0.247 |
| Meat and poultry, processed | 30.0 [12.9-59.3] | 26.7 [12.9-44.3] | 0.228 |
| Poultry, unprocessed | 16.7 [0.0-43.8] | 8.3 [0.0-29.3] | 0.158 |
| Soups | 25.0 [0.0-83.3] | 0.0 [0.0-54.4] | 0.143 |
| ^1^Data are presented as median [IQR], n=45. Differences were assed using a Wilcoxon signed rank test. | | | |
