# Supplementary material for: The effect of high compared with low dairy consumption on glucose metabolism, insulin sensitivity, and metabolic flexibility in overweight adults: a randomized crossover trial
Source: Am J Clin Nutr. 2019 Apr 17;109(6):1555–68. doi: 10.1093/ajcn/nqz017 (PMC6537937; doi:10.1093/ajcn/nqz017)
Supplement: nqz017_Supplemental_Files [file nqz017_supplemental_files.zip › OSM-Supplemental figure 1.doc]

**CONSORT 2010 Flow Diagram**

**Allocation**

**Analysis**

**Follow-Up**

**Enrollment**

Assessed for eligibility (n=63)

Excluded (n=10)

♦  Not meeting inclusion criteria (n=6)

♦  Declined to participate (n=2)

♦  Other reasons (n=2)

Analysed (n=22)
♦ Excluded from analysis (give reasons) (n=0)

♦ Lost to follow-up (n=0)

♦ Discontinued intervention (n=4)

-End of 1^st^ period: Claustrophobia, bronchitis, unable to continuously draw blood

-End of 2^nd^ period: sick after OGTT

♦ Replacement of drop-out (n=1)

Allocated to intervention order ‘High-Low’

(n=26)

♦ Received allocated intervention (n=25)

♦ Did not receive allocated intervention (n=1)

-Switched to ‘Low-High’ group before start due to practical reasons

♦ Lost to follow-up (n=0)

♦ Discontinued intervention (n=3)

-End of 1^st^ period: sick after OGTT (n=2), unable to continuously draw blood

Allocated to intervention order ‘Low-High’

(n=26)

♦ Received allocated intervention (n=26)

♦ Switched to intervention in this order (n=1)

♦ Did not receive allocated intervention (n=0)

Analysed (n=23)
♦ Excluded from analysis (give reasons) (n=1)

-gastro-intestinal illness before final test day

Randomized (n=52)
